# Immunoinformatics Strategy to Develop a Novel Universal Multiple Epitope-Based COVID-19 Vaccine

**DOI:** 10.3390/vaccines11061090

**Published:** 2023-06-12

**Authors:** Nizar A. Khamjan, Mohtashim Lohani, Mohammad Faheem Khan, Saif Khan, Abdullah Algaissi

**Affiliations:** 1 Department of Medical Laboratories Technology, College of Applied Medical Sciences, Jazan University, Jazan 45142, Saudi Arabia; 2 Department of Emergency Medical Services, College of Applied Medical Sciences, Jazan University, Jazan 45142, Saudi Arabia; 3 Medical Research Centre, Jazan University, Jazan 45142, Saudi Arabia; 4 Department of Biotechnology, K.C.M.T., M. J. P. Rohilkhand University, Bareilly 243006, India; 5 Department of Basic Dental and Medical Sciences, College of Dentistry, Hail University, Hail 2440, Saudi Arabia

**Keywords:** SARS-CoV-2, vaccine design, nucleocapsid, B cell, T cell

## Abstract

Currently available COVID vaccines are effective in reducing mortality and severity but do not prevent transmission of the virus or reinfection by the emerging SARS-CoV-2 variants. There is an obvious need for better and longer-lasting effective vaccines for various prevailing strains and the evolving SARS-CoV-2 virus, necessitating the development of a broad-spectrum vaccine that can be used to prevent infection by reducing both the transmission rate and re-infection. During the initial phases of SARS-CoV-2 infection, the nucleocapsid (N) protein is one of the most abundantly expressed proteins. Additionally, it has been identified as the most immunogenic protein of SARS-CoV-2. In this study, state-of-the-art bioinformatics techniques have been exploited to design novel multiple epitope vaccines using conserved regions of N proteins from prevalent strains of SARS-CoV-2 for the prediction of B- and T-cell epitopes. These epitopes were sorted based on their immunogenicity, antigenicity score, and toxicity. The most effective multi-epitope construct with possible immunogenic properties was created using epitope combinations. EAAAK, AAY, and GPGPG were used as linkers to connect epitopes. The developed vaccines have shown positive results in terms of overall population coverage and stimulation of the immune response. Potential expression of the chimeric protein construct was detected after it was cloned into the Pet28a/Cas9-cys vector for expression screening in *Escherichia coli.* The developed vaccine performed well in computer-based immune response simulation and covered a diverse allelic population worldwide. These computational findings are very encouraging for the further testing of our candidate vaccine, which could eventually aid in the control and prevention of SARS-CoV-2 infections globally.

## 1. Introduction

COVID-19, the most devastating infectious disease of the twenty-first century, has spread across the globe, culminating in countless deaths and enormous financial and social ramifications on a global scale [1]. The emergence of multiple variants of this lethal virus during 2021 was a crucial occurrence that added to the complexity of the current pandemic. There is a probability that COVID-19 will one day become endemic in the human population across the world [2]. This would imply that this virus is likely going to remain and live alongside humankind. The effectiveness and persistence of natural immunity concerning protection against SARS-CoV-2 reinfections and chronic conditions are of essential relevance for the future. This is because significant numbers of patients are continuing to be infected with the virus. Multiple variants of concern (VOC) and variants of interest (VOI) have been identified. Some of the mutants, including Alpha, Beta, Gamma, Delta, and Omicron, are VOC due to their increased transmissibility, decreased neutralization by the antibodies produced by an earlier infection or vaccination, decreased response to treatment, or to diagnostic failures. In contrast, VOI include M, Zeta, Lambda, and R1 (B.1.427/B.1.429) and others that display changes in receptor binding but not elevated hospitalizations and mortality [3]. Previous research has found that the vast majority of CD4 and CD8 T cell epitopes were completely preserved in the aforementioned variants, that T cells from exposed donors or vaccines accurately identified SARS-CoV-2 variants, and that the vast majority of the T cell response was unaffected [4].

The majority of the available vaccines are spike protein based, which has undergone numerous mutations in SARS-CoV-2 variants despite being highly immunogenic, allowing vaccine-generated immune responses to escape. The spike protein is a prominent surface antigen involved in virus entry into the host cell; hence, it is the target of neutralizing antibodies [5]. Data reported to date on the efficacy of different available vaccines has shown promising results in limiting the spread of infections. More than one hundred vaccines that can be taken orally or nasally are now under development across the world. The immunogenicity of the SARS-CoV-2 vaccine varies from individual to individual, and immune responses correlate with vaccination efficacy [6]. Several ‘universal’ vaccines are still in the development process. By incorporating the most immunogenic sequences of one or more pathogenic antigens, multi-epitope protein vaccines maximize vaccination potency. To optimize cross-efficacy, a multi-variant, multi-epitope vaccine encodes the most immunogenic conserved epitopes observed in all pathogenic strains [3].

Despite significant progress in the pharmaceutical field, no single drug completely cures this disease [7]. This necessitates the development of vaccines to target viruses at the molecular level. However, some progress has been made in the field of vaccine development. The promising vaccine’s role would be to activate and initiate herd immunity [8]. The packaging of the coronavirus positive-sense RNA genome requires nucleocapsid (N) proteins to form ribonucleoprotein complexes enclosed within the viral capsid. Due to its high antigenic potential and conserved properties, we selected the ‘N protein’ for this particular vaccine design to create a novel multi-epitope vaccine. The N forms a complex with genomic RNA, which in turn interacts with viral membrane proteins during virion assembly [8]. These are thought to be essential for improving the effectiveness of viral transcription and assembly. Innate immunity serves as the body’s initial line of defense against invasive pathogens [9]. TLR-3 receptors serve as sentinels for the innate immune system. TLR-3 is involved in the regulation of microbial entrance into the host as well as the immunological response [10]. Therefore, TLR-3 can be a good receptor for the proposed vaccine to analyze the generated immune response by the host. Using state-of-the-art in silico techniques, we have identified various potential vaccine constructs that may prove useful in the future for battling the pandemic. 

## 2. Materials and Procedures

The beta coronavirus N proteins were chosen for this study because of their high antigenic potential, capacity for invasion, and capacity for viral genome assembly. This protein may be an excellent candidate for a vaccine because it is involved in how viruses function. It does not specifically resemble any proteins identified in humans. Additionally, its subcellular localization was also revealed to be appropriate for vaccine development.

### 2.1. Sequence Retrieval, Structural Analysis, and Sequence Alignment

The complete genome of SARS-COV-2 was retrieved from the NCBI through the NC_045512 accession ID. The Sars-COV2 linear genome assembly produced using Illumina sequencing technology is available under NC_045512 accession. The assembly has a 29,903-bp ssRNA sequence. It has been identified that the majority of the recently published manuscripts refer to NC_045512. This assembly was sequenced under the PRJNA4854 genome project. The N protein was chosen as one of the antigenic proteins from the NCBI database, and multiple sequence alignment (MSA) of N-protein among the various variants of SARS-COV2 was performed by Clustal-w. Protein ideograms of the alignment were developed by using the karyoploteR module of the R-Bioconductor [11].

### 2.2. B-Cell Epitopes Prediction

The IEDB linear epitope prediction tool v2.0 (http://www.cbs.dtu.dk/services/BepiPred/ accessed on 15 January 2023) with default parameters was used to predict B-cell epitopes [12]. The software uses only epitope data from crystallized structures and is based on a complex algorithm based on antigen–antibody protein structures. As a result, it is recognized as a high-quality, precise, and powerful tool when compared with others [13].

### 2.3. T-Cell Epitopes Prediction 

MHC-I binding of conserved epitopes was predicted by the IEDB MHC-I binding prediction tool (http://tools.iedb.org/mhci accessed on 20 January 2023). The sequence prediction method was based on SMM in FASTA format. The host species was set as “human”. Only alleles with a length of 9 were selected. The output format was kept in XHTML, and all other options and parameters were left at their default settings. The IEDB MHC-II binding prediction tool (http://tools.iedb.org/mhcii accessed on 20 January 2023) was used to predict the MHC-II binding of conserved epitopes. Here also, the sequence was presented in FASTA format, and the prediction method was based on SMM. All human HLA-DR, HLA-DQ, and HLA-DP species/loci were set, and all alleles were selected at the default length parameter [14].

### 2.4. Allergenicity and Antigenicity Profiling of Selected T and B-Cell Epitopes

The VaxiJen 2.0 server was used to test the antigenicity of selected T- and B-cell epitopes [15]. This is based on the physiochemical properties of an alignment-independent server protein. The FASTA sequence provided as a parameter to the server was set as the default value. Allergenic testing of T and B-cell epitopes has been implemented using Allertop [16]. In addition, for the analysis of toxicity, the ToxinPred server was also used.

### 2.5. Analysis of Conservation

The IEDB’s Population Coverage Analysis tool (http://tools.iedb.org/population accessed on 22 January 2023) was used to detect allelic conservation and their coverage throughout the world. The number of epitopes was manually filled in the tool and all of the regions of the world were selected as passed parameters in the tool [17]. 

### 2.6. Multi-Epitope Vaccine Construction

All the selected B and T cell epitopes were incorporated to develop a multi-epitopic construct with the help of an adjuvant 50S ribosomal protein L7/L12 (UniProt ID: P9WHE3) sequence along with EAAAK, GPGPG, and AAY as the three linkers to assemble the whole vaccine construct. Thereafter a 6× His tag was also inserted at the C-terminal of the developed construct. Finally, the secondary and tertiary structures were predicted using Psipred and Rosetta web servers [18,19].

### 2.7. Analysis of Solubility and Physiochemical Properties

Solubility analysis was used to determine the quantitative purity of a material. The ExPASy-Protparam tool was used to analyze the physiochemical properties of the multiepitope vaccine construct [20], and SOLpro was used to analyze the vaccine’s solubility [21].

### 2.8. Extrapolation of Secondary and Tertiary Structures

The secondary and tertiary structures of the multiepitope vaccine construct were extrapolated using PsiPred [22] and RaptorX [23], respectively. Both tools provide information about the primary helix, plates, and coils in the relevant protein. 

### 2.9. Validation and Tertiary Structure Improvement

Through the GalaxyRefine server, the tertiary structure of the tested protein was verified and improved, it is one of the most reliable tools for the refinement of tertiary structures [24]. Side chains were initially rebuilt and repacked as part of the refinement process. The ensuing overall structural relaxation was then achieved by molecular dynamics simulation techniques. 

### 2.10. Docking Evaluation

Analysis of Protein–protein interaction was performed using the Hdocklite standalone tool for docking analysis. TLR3 (PDB ID: 2A0Z) was employed as a receptor for the predicted vaccine construct because it is known that tool-like receptors play a significant role in the initiation and boosting of an innate immune response [25].

### 2.11. Molecular Dynamic and Simulation Analysis of Predicted Vaccine Construct

Utilizing the online tool iMODS, a molecular dynamics analysis was performed to explain the typical protein motion within intrinsic coordinates using normal mode analysis [26]. This is founded on an examination of the complex’s torsional angles. The RMSD values, the covariance between individual residues, the Eigenvalue of interacted residues, and the deformation of the structure were all examined using this tool. It determines the stability of the complex based on a thorough analysis of the coordinates.

### 2.12. Codon Optimization of Designed Vaccine Peptide for Expression Analysis

Reverse transcription followed by codon optimization was performed for the vaccine construct by backtranseq and jcat server [27]. Thereafter the optimized vaccine construct was used for in silico cloning expression in Escherichia coli (*E. coli*-K12 strain), by using Snap gene software.

### 2.13. Analysis of Immune Simulation

An immunological simulation was undertaken using an online C-ImmSim server to make sure that the immune response was correct [28]. The server used a position-specific scoring matrix to identify immunological epitopes and their immune interactions.

## 3. Results

### 3.1. MSA Analysis and Selection of Conserved Segment for Consideration of Epitopes

The MSA analysis showed seven significant mutations between distinct variants. When choosing segments for the prediction of epitopes for the B-cell and MHC classes, mutation sites were excluded. Site 03, where aspartic acid changes from leucine in the Beta, Gamma, and Omicron variants, was the location of the initial mutation. At position 13, a conserved proline was switched out for a leucine, resulting in the second mutation. In the instance of Omicron, the third mutation was the largest deletion of E-R-S, amino acids at positions 31–33. Proline was substituted for arginine in the Delta and Omicron lineage, which was the fourth mutation. The fifth, sixth, and seventh mutations, on the other hand, involved the substitution of glycine for arginine (in Beta, site 80), Phenylalanine to Serine (in Beta, Gamma, and Omicron, site 235), and Serine to Arginine (in Omicron, site: 413) (File S1). 

**Figure 1:**
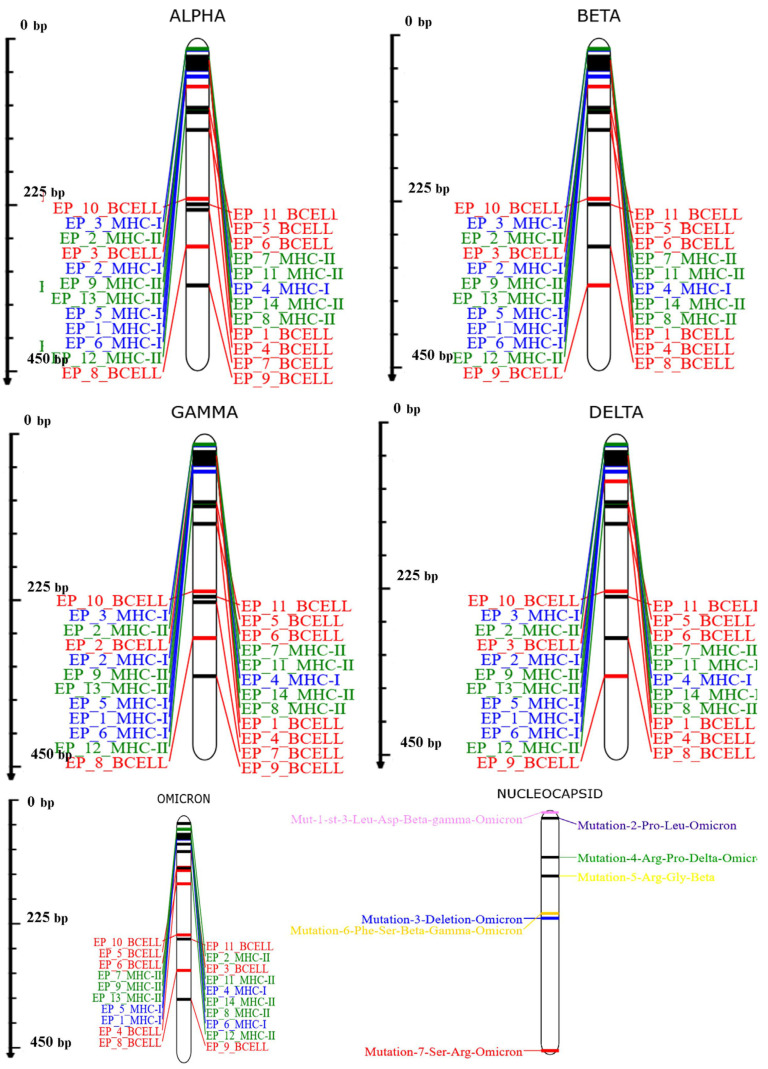
Epitope coverage in various major variants of SARS-CoV-2.

All the selected epitopes (B-cell, MHC class-I, MHC class-II) were observed to be best fitted in all variants of SARS-COV-2. All 20 predicted epitopes of MHC class-I and II covered approximately all the variants of the SARS-CoV-2 virus. Whereas 9 out of 11 linear epitopes of B-cell also covered all the variants of the same virus (Figure 1).

### 3.2. Sequence and Structure Analysis

One hundred sequences of the SARS-CoV-2 virus’s N-protein were extracted in order to build a potentially broad-spectrum vaccine against it. It is well known that infected cells can produce large amounts of N-proteins, which perform a variety of tasks, including binding to viral RNA to create the ribonucleocapsid. Additionally, it has been suggested that it might be involved in the replication, transcription, and translation of viruses. P0DTC9 (NCAP SARS2), an antigenic and highly effective viral protein, is present in UniProtKB. The VaxiJen 2.0 server calculated the antigenicity of viral proteins (File S2). The cutoff value was selected at 0.4 to ensure the validity of the test. Analysis of the full-length protein’s antigenic composition revealed that it might be a potent viral antigen (File S2).

### 3.3. Physiochemical Analysis, Secondary Structure and Transmembrane Topology Prediction of N Protein

**Figure 2:**
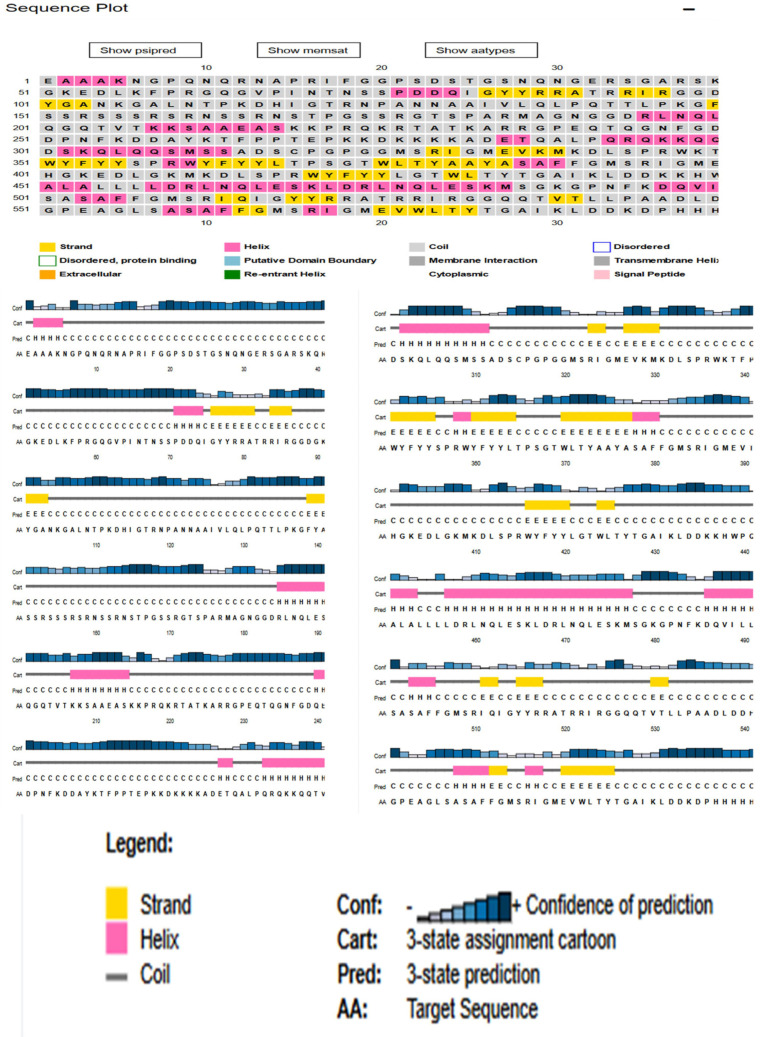
Secondary structure profiling of N-protein. The yellow segment shows the Beta strand, Pink: Helix, and Grey: Coiled structure.

The physical and chemical parameters of the protein measured using ProtParam, yielded a molecular weight of 45,625.70 Dalton for this protein composed of 435 amino acids. The computed isoelectric point (PI) value of 10.07 indicates a positive signal message. The instability index of 45.09 suggests that the selected protein is stable. Furthermore, the aliphatic index of 52.53 suggests that the protein is thermostable over a wide temperature range. The formula C1971H3137N607O629S7 represents the total number of carbon (C), hydrogen (H), nitrogen (N), oxygen (O), and sulfur (S) (Figure S1). According to PSIPRED and Rosetta, the N-protein has a 20% helix, a 12% sheet, and a 68% loop also shows the prediction of 25 disulfide bonds (S–S) using the ‘Disulfide by Design’ server (Figure 2) [29]. The transmembrane topological profile was predicted using the online program HMMTOP. The expected positions of the four transmembrane helices were 364–381, 41–431, 498–515, and 546–565. The total entropy of the best model was calculated to be 17.0182, while the entropy of the best path was calculated to be 17.0195 (Figure S2).

### 3.4. Linear B Cell Epitope Prediction

A hidden Markov model-based technique, used by Bepipred, is one of the most effective ways to predict linear epitopes. It is well known that B-cell epitopes play significant roles in the defensive mechanisms against viral infections. Potential B-cell epitopes play a crucial role in the direct recognition of B-cells and the activation of a variety of immune responses against particular viral infections. Here, we used techniques that rely on the screening of amino acids to explore and anticipate probable B-cell epitopes. We used a consensus-based approach with a threshold of 0.50 in the BePipred Linear Epitope Server for the prediction of 11 total cell linear epitopes by compilation in order to identify possible B-cell epitopes (Table 1). The score range for linear epitope prediction was 0.297 to 0.764. Furthermore, the average prediction score was determined to be 0.297. 

**Figure 3:**
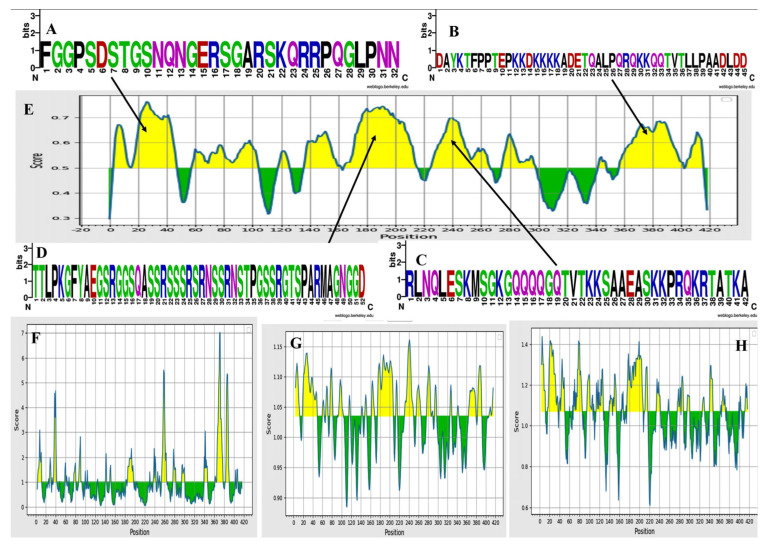
Characterization of predicted B-cell linear epitopes. (**A**–**D**): Colorful sequence logos showing highly conserved and populated predicted B-cell linear epitopes, (**E**): Bepipred linear epitope prediction of the nucleocapsid protein, (**F**): Karplus and Schulz flexibility prediction for the flexibility of nucleocapsid protein, (**G**): prediction of beta turns in nucleocapsid protein by using Chou and Fasman beta turn analyzing algorithm. (**H**): Emini surface accessibility prediction of nucleocapsid protein.

After thoroughly evaluating the data, we found that peptide sequences ranging in length from 17 to 48 and 161 to 216 amino acids can expedite the desired immunological response and are therefore recognized as B-cell epitopes for our developed vaccine construct (Table 1). The explanations of the outcomes of this approach are provided in Figure 3E, wherein the sequence logos depict the four highly conserved and populated epitopes throughout the world (Figure 3A–D). The Kolaskar and Tongaonkar approach was used to assess the antigenicity of experimentally identified amino acid epitopes as depicted in Table 2. The maximum antigenicity tendency was 1.240, while the minimum value was 0.875. The linear epitope prediction score ranged from 0.297 to 0.764. Additionally, it was observed that the average prediction score was 0.297. The peptide sequences with lengths of 17 to 48 and 161 to 216 amino acids were shown to most quickly elicit the necessary immunological response, and as a result, they were identified as B-cell epitopes after a thorough analysis of the data shown in Figure 3F. Antigenicity ranged from 1.240 at the greatest to 0.875 at the minimum.

The surface-exposed feature, hydrophilic nature, and beta-turn are known and are crucial for the start of the immune system’s defensive reaction. The beta-turn evaluation technique developed by Chou and Fasman was utilized to predict the beta-turn in N-protein. The calculated results recommended various values between 0.410 (minimum) to 1.439, based on a 1.070 threshold level and a mean value of 0.915. It was discovered that the peptide structure’s beta turns are more likely to be persuaded by the region from 196 to 202 (Figure 3G).

Experimental data indicated a connection between the peptides’ flexibility and the protein’s antigenicity. The Karplus and Schulz approach was developed as a result. This prediction method revealed that the area between 238 and 244 was more flexible, as seen in Figure 3F. The tool’s threshold value was changed to 1.035, and the computed results are 0.885 (the minimum) and 1.161 (the maximum). The calculated average value was 1.035. The epitopes were further sorted based on their antigenicity, allergenicity, and toxicity. Selected epitopes’ antigenicity was evaluated using a 0.4 threshold value. Only non-toxic and nonallergic epitopes were then used for future investigations. Eleven epitopes were considered to be efficient B-cell epitopes, capable of evoking B-lymphocytes in a highly enhanced manner (TTLPKGFYAEGSRGGSQASSRSSSRSRNSSRNSTPGSSRGTSPARMAGNGGD, GGPSDSTGSNQNGERSGARSKQRRPQGLPNN, RLNQLESKMSGKGQQQQGQTVTKKSAAEASKKPRQKRTAT, DAYKTFPPTEPKKDKKKKADETQALPQRQKKQQTVTLLPAADLDD, KADETQALPQRQKKQQTVTLLPAADLDD, SKQLQQSMSSADS). Using the IEDB conservancy analysis tool, B-cell epitopes were further examined for conservancy analysis. A total of 31 epitopes, including B cells, MHC class I, and MHC class II, were chosen for use in the creation of the vaccine after being employed in conservation analysis. These epitopes were discovered to be conserved with maximum conservation (from 96% to 100% coverage and identity) in more than 90% of the epitopes (File S3).

### 3.5. T-Cell Epitope Identification

#### 3.5.1. Prediction of MHC Class-I Binding Profile for Conserved Epitopes

We chose to study a wide range of MHC–HLA alleles in humans using the SMM approach and the homo sapiens MHC source. This utility offers an output interface for epitope HLA-binding affinity in nM IC50 units. A stronger binding affinity of epitopes to MHC Class-I molecules is indicated by a lower IC50 value. Based on IC50 values less than 100, a total of 141 epitopes were chosen, each of which was predicted to interact with a large number of MHC-Class-1 alleles. Based on the highest level of MHC-Class-1 allele interaction with the 141 epitopes, 68 were chosen. Forty-three epitopes were further filtered based on antigenicity, allergenicity, and toxicity. Epitopes that were toxic or allergenic and had antigenic scores of less than 0.4 were not included. MHC Class-1’s finalized 06 epitopes were prepared for further investigations. The core epitopes GMSRIGMEV, KMKDLSPRW, KTFPPTEPK, LSPRWYFYY, SPRWYFYYL, and TPSGTWLTY, were perceived to be the dominant binders with 20 alleles (HLA-A*02:03, HLA-A*02:01, HLA-A*32:01, HLA-B*58:01, HLA-B*57:01, HLA-A*11:01, HLA-A*30:01, HLA-A*03:01, HLA-A*31:01, HLA-A*68:01, HLA-B*53:01, HLA-B*35:01, HLA-A*30:02, HLA-A*01:01, HLA-A*68:02, HLA-A*02:06, HLA-B*07:02, HLA-B*08:01, HLA-B*35:01, HLA-B*53:01). The core epitope KMKDLSPRW showed the highest antigenic score, 1.7462 (Table 3).

**Figure 4:**
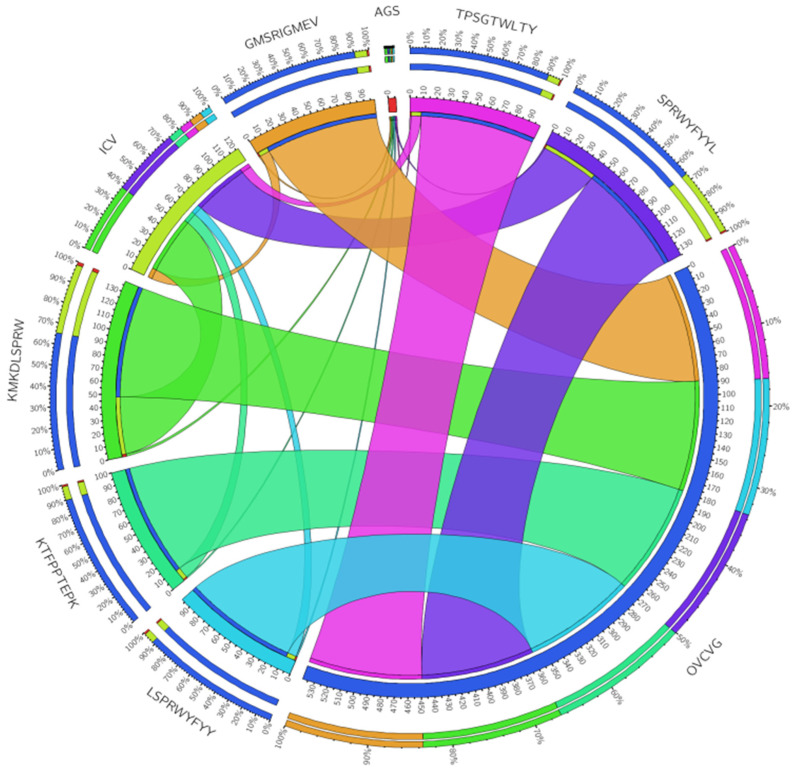
Characterization of Class-I MHC conserved epitopes with respect to their ICV value, population coverage and distribution throughout the world.

CIRCOS graphical view summarizes the distribution of six core MHC class-I epitopes along with their antigenicity value, ICV value, and overall coverage throughout the world. The first and second tracks show the compact view of all parameters taken in the study for sorting MHC-class I epitopes (value, ICV value and overall coverage). The third track indicates the size of each epitope. A wide ribbon indicates a high value whereas a thinner feature suggests a lower value. The ribbon connection from one node to another node shows the relation between parameters. In the CIRCOS figure, indigo represents SPRWYFYYL, magenta shows TPSGTWLTY, orange shows GMSRIGMEV, green shows KMKDLSPRW, mint shows KTFPPTEPK, and the sixth class I epitope is shown by the dark cyan shade color (Figure 4).

#### 3.5.2. MHC Class II Binding Profile Prediction for Conserved Epitopes 

It was found that 1217 predicted conserved epitopes with IC50 values under 100 interacted with MHC Class-II alleles. Thirty-two of the 1217 epitopes that were interacting with more than six MHC Cass-II alleles were chosen (Table 4). Due to their allergenicity, toxicity, and antigenicity, 11 epitopes were chosen for further examination. As it interacts with 131 alleles, the core epitope LALLLLDRLNQLESK is thought to be the top binder, followed by ASAFFGMSRIGMEVT and QVILLNKHIDAYKTF, which are predicted to bind with 100 and 99 alleles, respectively (File S2).

### 3.6. Assembly of Vaccine Construct

**Figure 5:**
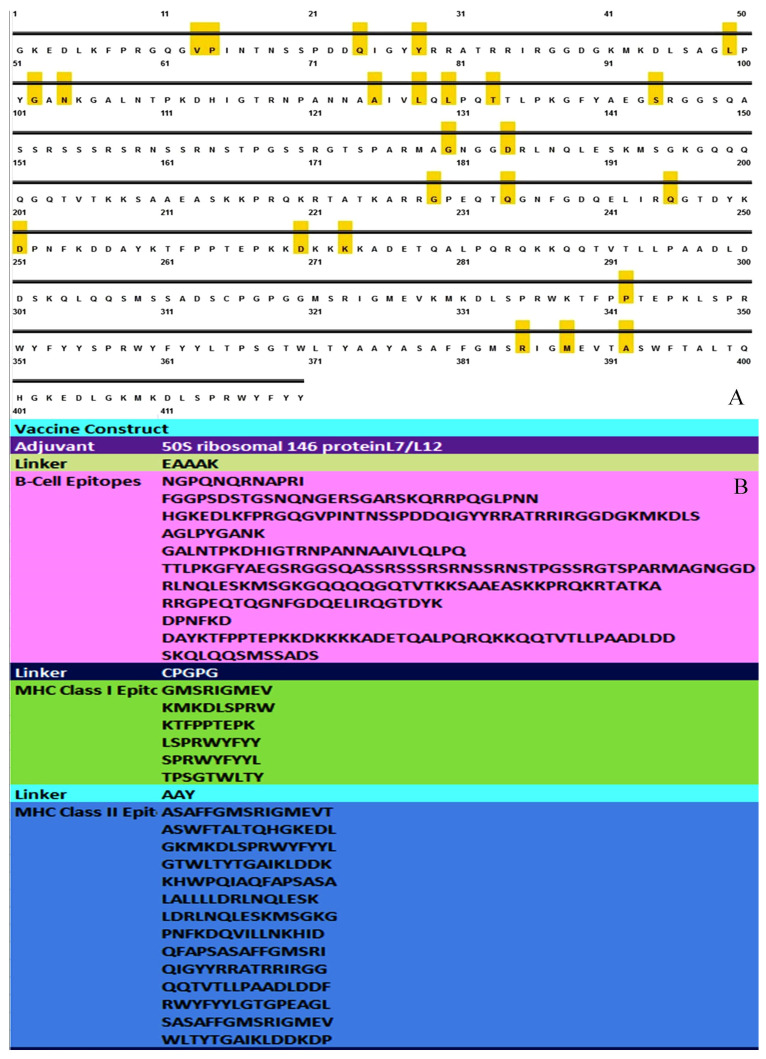
Disulfide characterization and multi-epitopic assembly of the constructed vaccine. (**A**) Disulfide characterization of the constructed vaccine. (**B**) Multi-epitope assembly of the constructed vaccine.

A total of 11 B-cell epitopes, 06 MHC Class-I epitopes, and 14 MHC Class-II epitopes were employed to create the multi-epitopic vaccination chimera. In order to produce the vaccine, the 50S ribosomal protein L7/L12 with the UniProt ID P9WHE3 was employed as an adjuvant. To elicit a particular immunological response, the adjuvant was joined to the first B-cell epitope using an EAAAK linker at the amino (N) terminus. Additionally, GPGPG linkers were used to link B-cell and MHC Class-I epitopes. AAY linkers were used to connect MHC Class-II epitopes. To minimize vaccine size, overlapping B-cell, CTL, and HLT epitope areas were combined. (Figure 5). At the C-terminus of the vaccine sequence, a 6× His tag was added for the protein recognition and separation phase. The molecular weight of the ultimate vaccine construct sequence was 65,889.99 (Figure S4).

### 3.7. Investigation of the Population Coverage and Epitope Conservation

A population coverage study was carried out to determine the global coverage of MHC Class-I and MHC Class-II allele interaction epitopes. The most prevalent candidate epitopes for each coverage approach were identified using the IEDB population coverage analysis tool. The MHC HLA allele distribution fluctuates across a variety of geographical locations worldwide. A population coverage study was carried out to determine the global coverage of MHC Class-I and MHC Class-II allele interaction epitopes. The most prevalent candidate epitopes for each coverage approach were identified using the IEDB population coverage analysis tool. The MHC HLA allele distribution fluctuates across a variety of geographical locations worldwide. To build a potential vaccine, population coverage is therefore required. The regions with the highest population exposure for the MHC Class-II allele were Europe (97.90%), North America (94.70%), the West Indies (91.20%), South Asia (90.95%), West Africa (89.04%), North Africa (87.36%), Northeast Asia (87.09%), Southwest Asia (85.05%), South America (83.55%), Southeast Asia (79.12%), East Asia (78.56%), East Africa (76.90%), Oceania (73.17%), South Africa (70.90), Central Africa (70.07), and Central America (37.17%). Central America had the lowest population coverage calculated (37.17%). Black South Africans, however, had the lowest population coverage calculated (2.58%). Six epitopes (GMSRIGMEV, KMKDLSPRW, KTFPPTEPK, LSPRWYFYY, SPRWYFYYL, and TPSGTWLTY)—representing a large coverage in contrast with the global population—account for the majority of interactions between MHC Class-I alleles. It was estimated that 90.07% of these epitopes would be covered by concentrated populations worldwide. In the case of MHC Class-II, 11 epitopes were predicted to interact with frequent MHC Class-II alleles (ASAFFGMSRIGMEVT, ASWFTALTQHGKEDL, GKMKDLSPRWYFYYL, GTWLTYTGAIKLDDK, KHWPQIAQFAPSASA, LALLLLDRLNQLESK, LDRLNQLESKMSGKG, and PNFKDQVILLNKHID), with ASAFFGMSRIGMEVT receiving the highest population coverage percentage among these epitopes worldwide, with a score of 97.96%. The population coverage study outcomes for the numerous binders to MHC Class-I and MHC Class-II alleles, respectively and in combined form, are the most exciting aspect of this assessment. They show spectacular coverage with a percentage of about 90% and 96%, respectively. The conservancy was assessed using the IEDB’s conservancy analysis tool (File S3).

### 3.8. Analysis of Solubility and Physiochemical Properties of Multi-Epitope Subunit 

ExPASY Protparam was used to predict physiochemical properties, and the results offer several properties relevant to the nature of the protein. The multiepitope subunit’s molecular weight was 65,889.99 Da. The protein’s calculated pI was 9.99. According to this value, the protein may be of a basic type with an instability index of 59.80 (II). The aliphatic index values of 54.19 and the GRAVY index of 0.981 revealed that it is a thermo-stable protein. The protein is not hydrophilic, as indicated by the positive result. The solubility rate for our vaccine design, as determined by the protein–sol server, was greater than its score indicated (0.485) (Figure S4).

### 3.9. Antigenicity and Allergenicity Evaluation of the Vaccine Protein

Using the VaxiJen 2.0 web server, the antigenicity of the vaccination protein and adjuvant was estimated to be 0.5059. Without an adjuvant, the vaccine construct’s antigenicity was estimated to be 0.5840. The results show that the vaccine construct is antigenic by nature, whether an adjuvant is linked to it or not. According to the results of AllerTOPv2, the vaccine was confirmed to be non-allergenic whether an adjuvant is linked to it or not. Toxinpred determined that the constructed vaccine was non-toxic, whether it was given an adjuvant or not (File S2).

### 3.10. Secondary Structure Extrapolation

The Psipred tool, which examined the protein’s real makeup, was used to extrapolate the secondary structure. The protein’s composition was determined by the results to have a 20% helix, 12% beta strands, and 68% coils. A total of 46% of the protein content was discovered to be exposed, 24% to be moderately exposed, and 20% to be hidden. A total of 14% of the residues were found to be in the disordered domain (Figure S3).

### 3.11. Protein’s Tertiary Structure Evaluation

By using the TrRosetta, the first-best tertiary structure model of the chimeric vaccine construct was created. Using the top 10 threading templates, the models were projected based on high coverage values. The model with the highest coverage score was chosen for refining operations in this study.

### 3.12. Tertiary Structure Prediction and Validation of Vaccine Construct 

**Figure 6:**
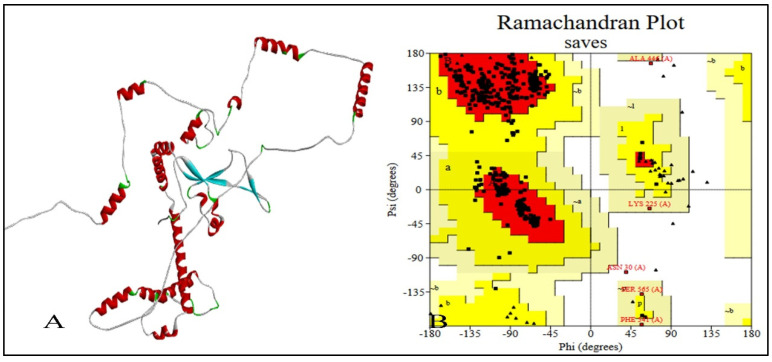
(**A**) Depiction of the predicted tertiary structure of constructed vaccine and (**B**) its amino acid distribution, categorized by Ramachandran plot.

The first-best tertiary structure model of the chimeric vaccine construct was constructed using the Rosetta. Using the top 10 threading templates, the models were projected based on high coverage values. The model with the highest coverage score was chosen for refining operations in this study. After refining, the Galaxy refine tool produced a total of five models of the chimeric vaccine. The refinement procedure took into account a number of variables, including GDT-HA (0.9519), RMSD (0.418), and Mol Probity (1.184). Ramachandran predicted a clash score of 3.5, a poor rotamer score of 0, and a forecasted Ramachandran score of 97.8%. Model 1 was chosen for later examinations since it was discovered to be the most authentic (Figure 6A,B).

The Procheck server verified the revised tertiary structure of the constructed vaccine. Ramachandran plot of the constructed vaccine depicted the significant changes before and after the refinement process of the structure. Before refinement, 91% of the region was in the plot’s preferred area, but just 8.4% of the structural region was present in the permitted area. Only 0.9% was in the outlier region whereas better results were obtained by Procheck after the refinement. After refinement, 93% of residues were updated in the preferred zone, 6.1% in the permitted region and 0.4% in the outlier region, as observed in Figure 6B.

### 3.13. Molecular Docking with Ligand Binding Domain of TLR3

**Figure 7:**
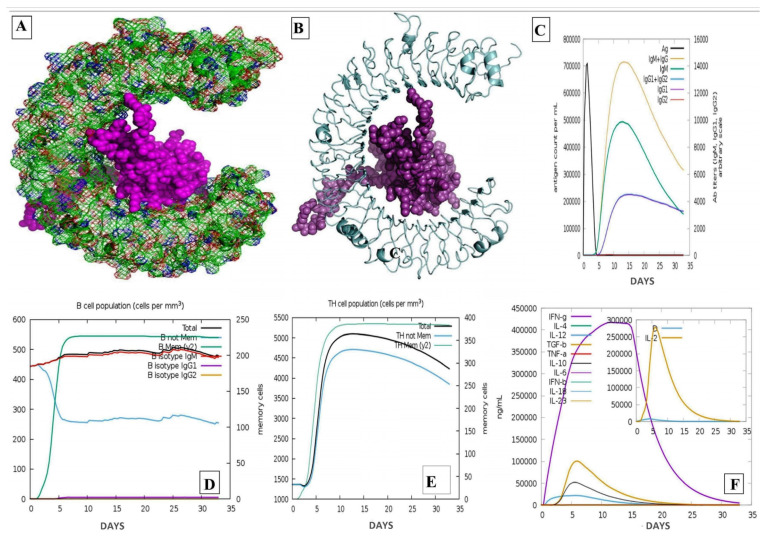
Protein–protein docking between constructed vaccine and receptor (TLR3) and their immune stimulation. Visualization of the docking between the epitope (**A**) A cartoon representation of the docking complex, (**B**) a docking visualisation of the interaction between the vaccine and its TLR3 receptor. (**C**) The generation of immunoglobulin following antigen injection; (**D**) the number of B cells following three injections. (**E**) The Helper T Cell Population by State, (**F**) Production of Cytokines and Interleukins with the Immune Simpson Index.

Using HDOCK software [30], protein–protein docking was carried out to predict the interaction between the refined vaccine model and the ligand binding region of the immunological receptor TLR3. After analyzing all 10 docked poses, model number 1 proved to be the best-docked model having 11 hydrogen bonds and 5 electrostatic interactions with a score of −398.38 as shown: A:ARG331, A:ASN229, A:ASN285, A:ASN287, A:ASN308, A:ASN309, A:ASN61, A:ASP153, A:ASP280, A:GLN107, A:GLU175, A:GLU434, A:GLU533, A:HIS108, A:HIS156, A:HIS39, A:HIS410, A:HIS60, A:ILE411, A:LEU177, A:LYS200, A:LYS201, A:LYS335, A:LYS382, A:MET131, A:MET278, A:PHE227, A:PHE333, A:PHE84, A:SER132, A:SER155, A:SER254, A:THR334, A:TYR283, A:TYR307, A:TYR383, A:TYR462, A:VAL435, B:ALA103, B:ALA150, B:ARG145, B:ARG42, B:ARG59, B:ARG84, B:ARG86, B:ASN104, B:ASP89, B:GLN132, B:GLN149, B:GLN61, B:GLU142, B:GLY143, B:GLY60, B:GLY90, B:LEU130, B:LEU135, B:LYS105, B:LYS137, B:MET92, B:PRO58, B:SER144, B:SER148, B:SER155, B:THR134, and B:TYR140 (Figure 7). All the interaction types, which include contact surface area, H-bond, interface interaction, Pi-interactions, and salt bridge are provided in File S4.

**Figure 8:**
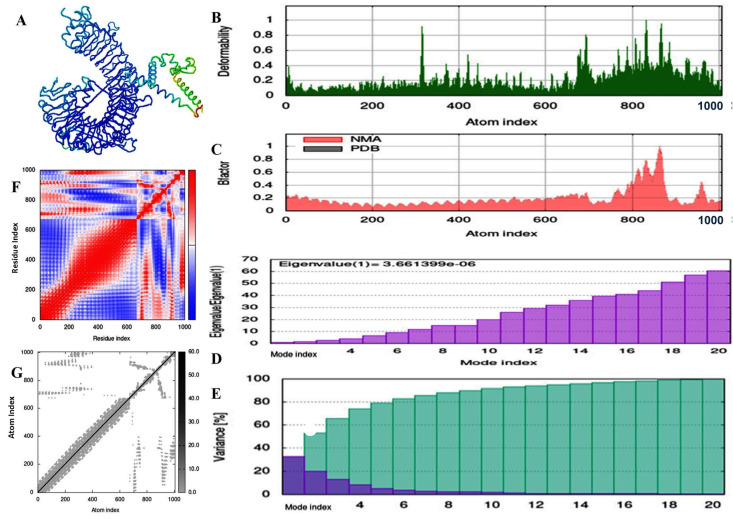
Molecular dynamics simulation analysis of vaccine–receptor complex. (**A**) Interaction visualization of the constructed vaccine and its TLR3 receptor, (**B**) Deformability, (**C**) B-factor plot, (**D**) Eigenvalue (**E**) Variance map, (**F**) Covariance map (**G**) Elastic network model.

IMOD adjusted the docking complex’s force fields several times using different time interval approaches and thereafter, the final less distorted best model was obtained as shown in Figure 8. The complex’s Eigen value was 3.661399 × 10^6^ (Figure 8D). Heat maps with low RMSD and highly correlated regions showed improved relationships between the individual residues. The supplied protein structure’s MNA mobility is depicted in Figure 8A, whereas the deformability portion of the Figure 8B demonstrates low levels of deformation at the entire residue.

### 3.14. Codon Optimization and Cloning Expression Analysis 

**Figure 9:**
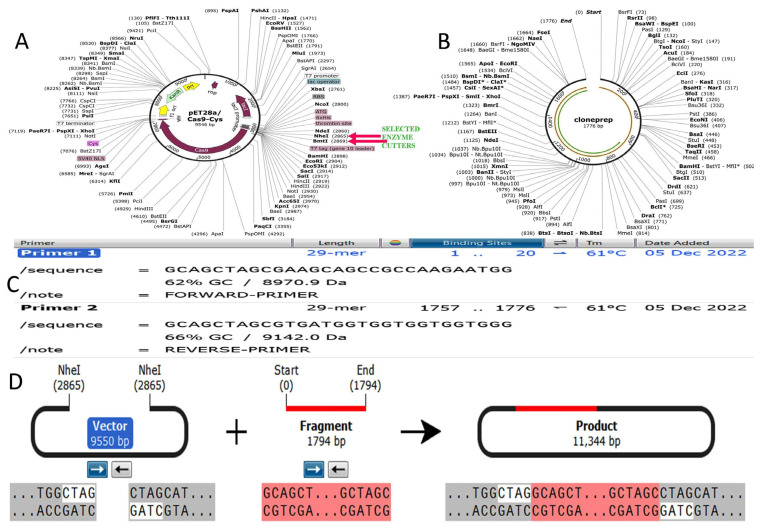
Restriction mapping of cloning vector and constructed vaccine for their compatibility in cloning expression by using Pet28a/Cas9-cys vector. Figure 9 explains the used cloning vector (**A**) Pet28a/Cas9-cys vector, (**B**) Clone prep means depicted the developed vaccine construct (**C**) Primer 1 and 2 depicts forward and reverse primers used for insilico PCR amplification (**D**) This segment shows the construction of chimeris product by fusion of vector and vaccine construct.

The amino acid sequence of the constructed vaccine was reverse transcribed through backtranseq program of EMBOSS 6.0.1 and thereafter the codons were optimized by using JCAT tool for better protein expression. The resulting optimized sequence showed CAI values 0.92 and 58.28% of GC content which further satisfied the stability for recombinant vector expression in E coli. Finally, the optimized sequence was cloned in pET28acas9/cys vector of 9550 bp along with 1794 bp of constructed vaccine sequence. An amount of 11,344 recombinant products formed that can be ready for cloning and expression. Insilco PCR using Snapgene also suggested the significance of the constructed vaccine as a very beneficial candidate (Figure 9).

## 4. Discussion

In this study, we focused on the N protein involved in the virus’s structural and pathogenesis activities. Evaluation of the protein’s physical and chemical properties indicates that it would make a potent vaccine. Since it is not available, the 3D protein structure for the N protein was modeled. Overall, this study demonstrates that multi-epitope-based subunit peptides can improve both humoral- and cell-mediated immune responses. B-cells were once thought to be the only source for future vaccine development. The most interfering human leukocyte antigen (HLA) strategy has, however, been used to target the major histocompatibility complex (MHC) T-cells, opening up a new field of clinical study [31]. With lag, antagonistic genetic drift can remove the antigen from the antibody’s memory. Although T-cell immunity offers a persistent immune response, the epitope must pass certain strict requirements to become a vaccine. The position of the polypeptide chains to be matched is related to characteristics such as bend, hydrophilicity, flexibility, the polarity of the exposed surface, accessibility, and antigenic tendency. The Karplus and Schulze and Bepipred linear epitopes, Emini surface, Chou and Fasman beta-turn, Kolaskar and Tongaonkar, and linear epitopes may all be analyzed computationally to determine which residues have the greatest potential to influence the evolution of the epitope. However, they also offer the peptide sequences of the epitopes for further examination. Here, we looked at possible T-cell epitopes that are highly active against their target allele and have an IC50 value of less than 100. Epitopes interacting with more than five MHC class-I and II molecules were isolated for further screening using the consensus approach. After demonstrating that they were the best among several criteria, the epitopes generate heated controversy. The investigation of both B and T cells’ antigenic properties is the most crucial of these. 

It was found by the MSA of various variants of SARS-COV2 that the N protein was less mutated except in Omicron, where some mutations and deletions were seen. Therefore, the conservation property of this N-protein was a major reason for selection in the current study. Because of the high conservancy of this protein, all three major epitopes of B-cell, MHC class-I, and MHC class-II were selected as best-fitted candidates for all variants of the studied virus from this protein.

A major barrier to the development of vaccines is allergy. Currently, the majority of vaccines cause allergic reactions in order to boost the immune system. The FAO/WHO allergenicity prediction scheme, however, states that a sequence is most likely allergenic if it contains at least six consecutive amino acids when compared with the database of known allergens [32]. Our chosen epitopes were therefore determined to be non-allergens by AllerTop v. 2.0 since they did not match the requirements of the FAO/allergenicity WHO’s prediction evaluation scheme. In our subsequent research, the probable antigenic epitopes free of allergenicity and toxicity were recognized as crucial for producing immunoreactive peptides. The selected epitopes are candidates for vaccine development since they are conserved across all HCoV strains. IEDB-filtered epitopes exhibit good conservation in protein sequence fraction, and identity is the level of similarity between strains. Additionally, the IEDB performed a population coverage study for T-cells because MHC molecules are incredibly polymorphic and can be found in thousands of different human MHC (HLA) alleles. For this reason, numerous T-cell peptides with various HLA bindings were examined. Globally, both MHC classes exhibit high conservation. The community of HCoV patients who are at risk from peptide-based vaccines will receive more coverage as a result. Following the fulfillment of all requirements, 11 epitopes of B-cell, six T-cell epitopes of MHC Class-I, and 14 MHC Class-II epitopes were chosen as subunits for the vaccine construct-building process. Since a study has revealed that the 50S ribosomal protein L7/L12 is involved in pathogen detection and immune system activation in enhancing the response to vaccines, it was utilized as an adjuvant to improve immunological qualities. To create the multi-epitope subunit vaccine design, specific B- and T-cell epitopes were chosen as the best linkers. Due to their high efficiency, spacer sequences are crucial to the processes involved in developing vaccines. To generate a prospective vaccination with maximum antigenicity, the GPGPG and AAY linkers were developed to integrate the complete vaccine design between projected epitopes. An EAAAK linker was included in the sequence design to connect the adjuvant to a previously anticipated B-cell epitope. It has also been claimed that this linker’s entanglement can be exploited to create dual-purpose peptides that improve joined proteins. The 6 His tag, also known as a polyhistidine tag, is located at the sequence’s carboxyl (C-) terminus and consists of at least six histidine residues. Even under buffered circumstances, the sequence proceeds more quickly and easily due to histidine residues’ ability to bind to stabilized ions. Immunological testing and bioinformatics analysis showed that the created protein sequence lacked poisonous and allergenic characteristics. The antigenicity of the vaccine formulation was shown to be of low value, according to several recorded investigations. However, this artificial vaccination chimaera expressed positive antigenic scores that either did not bind to the adjuvant or did so in a suitable manner. The developed vaccine protein’s molecular weight was determined to be 45,625.70 daltons, and its solubility was further examined in light of its stimulus antigenicity. 

The vaccine’s theoretical PI of 10.07 validates the natural origin of the vaccination protein. The vaccine’s volatility index is low (a 45.09 score), indicating that the expressed vaccine model can be employed and that the proposed vaccine protein is stable. Aliphatic index analyses suggested that the chimeric vaccine design would be thermostable. In the design of vaccines, secondary and tertiary structures are regarded as crucial. The results of 3D structure prediction and validation showed that only a small number of remains were found in the outlying region, while the majority were found in the more favorable regions. This has been discovered to represent a desirable model quality that is acceptable. It is well known that the stampede (from the Ramachandran plot) illustrates the necessary conditions for competent vaccine potential. 

Numerous investigations have revealed that TLR, TLR3 in particular, is involved in triggering an immunological response to SARS-CoV-2. The specificity of TLR3 in the operation of innate immunity has been described in a study. The docked complex’s least energy value reveals a stable connection and a lower RMSD than the starting conformation. Strong hydrogen bonds, van der Waals forces, electrostatic interactions, and hydrophobic interactions all contribute to the ligand’s stable conformation inside the receptor’s binding pocket [33]. Docking provides us with a single image of intricate physiological motion. Therefore, a more adaptable environment is required for the investigation of p–p interaction. A molecular dynamics simulation was run to achieve this goal, simulating the dynamic system’s typical behavior. 

The created complex is demonstrated to be stable and shows fewer chances of deformation during an immune response based on its maximal eigenvalue. The hinges of the structure play a major role in how the structures deform. The hinges that were present across the entire building did not seem critical and were stable [34]. The analysis of the B factor revealed no discernible ups and downs, indicating an extremely low loop number. Further evidence for stable vaccine–TLR3 receptor binding comes from these observations. By using covariance matrix analysis, it was possible to identify the immune simulation of the planned construct, and the results were consistent with the immune responses. It was predicted that the injection of the vaccine into the body would result in a humoral response. Although numerous potential vaccine candidates have been examined using in-silico methods, no vaccine has been successfully created using the N protein of a new coronavirus. Additionally, immunological simulation and vaccine cloning were not carried out when developing earlier vaccinations. Compared with the vaccine chimaera we created, several vaccine candidates have limited population coverage. Lastly, further research is required to demonstrate that this is a viable prospective vaccination candidate.

## 5. Conclusions

In this study, computational approaches were employed to successfully build an effective vaccine candidate against SARS-CoV-2. An in silico immunological simulation depicted the immune response concerning the antigen’s clearance. Protein expression was good after computational cloning with SnapGene onto the Pet28a/Cas9-cys plasmid. The final criterion to guarantee the efficacy of a vaccine formulation against COVID-19, though, is experimental validation. The idea of this vaccine’s creation can be taken into consideration because peptide vaccines have produced positive outcomes in numerous studies with improved immune responses. By creating a potent vaccination, this work will undoubtedly aid in the fight against, or elimination of, the global threat posed by COVID-19.

## Figures and Tables

**Table 1:** Linear B-cell epitope prediction using Bepipred.

Start	End	Peptide	Length
4	15	NGPQNQRNAPRI	12
17	48	FGGPSDSTGSNQNGERSGARSKQRRPQGLPNN	32
59	105	HGKEDLKFPRGQGVPINTNSSPDDQIGYYRRATRRIRGGDGKMKDLS	47
119	127	AGLPYGANK	9
137	163	GALNTPKDHIGTRNPANNAAIVLQLPQ	27
165	216	TTLPKGFYAEGSRGGSQASSRSSSRSRNSSRNSTPGSSRGTSPARMAGNGGD	52
226	267	RLNQLESKMSGKGQQQQGQTVTKKSAAEASKKPRQKRTATKA	42
276	299	RRGPEQTQGNFGDQELIRQGTDYK	24
343	348	DPNFKD	6
358	402	DAYKTFPPTEPKKDKKKKADETQALPQRQKKQQTVTLLPAADLDD	45
404	416	SKQLQQSMSSADS	13

**Table 2:** The Kolaskar and Tongaonkar method for the prediction of antigenicity.

Start	End	Peptide	Length
52	59	WFTALTQH	8
69	75	GQGVPIN	7
83	89	QIGYYRR	7
106	115	PRWYFYYLGT	10
119	124	AGLPYG	6
130	136	IIWVATE	7
154	166	NAAIVLQLPQGTT	13
217	227	AALALLLLDRL	11
243	249	GQTVTKK	7
267	273	AYNVTQA	7
299	315	KHWPQIAQFAPSASAFF	17
333	339	YTGAIKL	7
347	363	KDQVILLNKHIDAYKTF	17
379	385	TQALPQR	7
389	401	QQTVTLLPAADLD	13
403	411	FSKQLQQSM	9

**Table 3:** Core selected MHC-class-I, epitopes used in constructed vaccine candidate.

Peptide Start	Peptide End	IC50	Epitopes	Allergenicity	Antigenic Score	Antigenicity
36	44	7.43	GMSRIGMEV	HLA-A*02:03	0.6287	NA
30	38	46.84	KMKDLSPRW	HLA-A*32:01	1.7462	NA
11	19	8.51	KTFPPTEPK	HLA-A*11:01	0.7571	NA
34	42	44.28	LSPRWYFYY	HLA-A*30:02	1.2832	NA
35	43	8.33	SPRWYFYYL	HLA-B*07:02	0.734	NA
45	53	7.45	TPSGTWLTY	HLA-B*35:01	0.145	NA

(NA = non-allergic).

**Table 4:** Core MHC class II epitopes used in constructed vaccine candidates.

Peptide Start	Peptide End	IC50	Epitopes	Allergenicity	Antigenic Score
82	96	44.72	KHWPQIAQFAPSASA	non allergen	0.4293
127	141	45	LALLLLDRLNQLESK	non allergen	0.4293
311	325	70.51	QIGYYRRATRRIRGG	non allergen	0.4614
348	362	71.83	QQTVTLLPAADLDDF	non allergen	0.4614
311	325	70.51	QFAPSASAFFGMSRI	non allergen	0.4658
311	325	89.66	SASAFFGMSRIGMEV	non allergen	0.6584
219	233	54.34	LDRLNQLESKMSGKG	non allergen	0.7029
354	368	81.97	RWYFYYLGTGPEAGL	non allergen	0.7505
325	339	8.27	ASAFFGMSRIGMEVT	non allergen	0.862
82	96	69.21	PNFKDQVILLNKHID	non allergen	0.988
311	325	39.08	GTWLTYTGAIKLDDK	non allergen	0.9934
82	96	17.28	ASWFTALTQHGKEDL	non allergen	0.4116
348	362	38.67	GKMKDLSPRWYFYYL	non allergen	1.1625
349	363	95.13	WLTYTGAIKLDDKDP	non allergen	1.2787

## Data Availability

The data presented in this study are available on request from the corresponding author.

## Supplementary Materials

The following supporting information can be downloaded at: https://www.mdpi.com/article/10.3390/vaccines11061090/s1.

## References

[1] Feng W., Zong W., Wang F., Ju S. (2020). Severe acute respiratory syndrome coronavirus 2 (SARS-CoV-2): A review. Mol. Cancer.

[2] Li J., Jia H., Tian M., Wu N., Yang X., Qi J., Ren W., Li F., Bian H. (2022). SARS-CoV-2 and Emerging Variants: Unmasking Structure, Function, Infection, and Immune Escape Mechanisms. Front. Cell. Infect. Microbiol..

[3] Palatnik-de-Sousa I., Wallace Z.S., Cavalcante S.C., Ribeiro M.P.F., Silva J.A.B.M., Cavalcante R.C., Scheuermann R.H., Palatnik-de-Sousa C.B. (2022). A novel vaccine based on SARS-CoV-2 CD4^+^ and CD8^+^ T cell conserved epitopes from variants Alpha to Omicron. Sci. Rep..

[4] Tarke A., Grifoni A., Sette A. (2022). Bioinformatic and Experimental Analysis of T Cell Immune Reactivity to SARS-CoV-2 and its Variants. Front. Bioinform..

[5] Harvey W.T., Carabelli A.M., Jackson B., Gupta R.K., Thomson E.C., Harrison E.M., Ludden C., Reeve R., Rambaut A., Peacock S.J. (2021). SARS-CoV-2 variants, spike mutations and immune escape. Nat. Rev. Microbiol..

[6] Mentzer A.J., O’Connor D., Bibi S., Chelysheva I., Clutterbuck E.A., Demissie T., Dinesh T., Edwards N.J., Felle S., Feng S. (2022). Human leukocyte antigen alleles associate with COVID-19 vaccine immunogenicity and risk of breakthrough infection. Nat. Med..

[7] Mullard A. (2022). COVID antibody drugs have saved lives-so why aren’t they more popular?. Nature.

[8] Chang C.K., Hou M.H., Chang C.F., Hsiao C.D., Huang T.H. (2014). The SARS coronavirus nucleocapsid protein—Forms and functions. Antivir. Res..

[9] Pan Y., Cai W., Cheng A., Wang M., Yin Z., Jia R. (2022). Flaviviruses: Innate Immunity, Inflammasome Activation, Inflammatory Cell Death, and Cytokines. Front. Immunol..

[10] Lien E., Ingalls R.R. (2002). Toll-like receptors. Crit. Care Med..

[11] Link A. (2017). karyoploteR: An R/Bioconductor package to plot customizable genomes displaying arbitrary data. Bioinformatics.

[12] Jespersen M.C., Peters B., Nielsen M., Marcatili P. (2017). BepiPred-2.0: Improving sequence-based B-cell epitope prediction using conformational epitopes. Nucleic Acids Res..

[13] Vigan-Womas I., Spadoni J.L., Poiret T., Taieb F., Randrianarisaona F., Faye R., Mbow A.A., Gaye A., Dia N., Loucoubar C. (2023). Linear epitope mapping of the humoral response against SARS-CoV-2 in two independent African cohorts. Sci. Rep..

[14] Reynisson B., Alvarez B., Paul S., Peters B., Nielsen M. (2020). NetMHCpan-4.1 and NetMHCIIpan-4.0: Improved predictions of MHC antigen presentation by concurrent motif deconvolution and integration of MS MHC eluted ligand data. Nucleic Acids Res..

[15] Doytchinova I.A., Flower D.R. (2007). VaxiJen: A server for prediction of protective antigens, tumour antigens and subunit vaccines. BMC Bioinform..

[16] Dimitrov I., Flower D.R., Doytchinova I. (2013). AllerTOP—A server for in silico prediction of allergens. BMC Bioinform..

[17] Bui H.H., Sidney J., Dinh K., Southwood S., Newman M.J., Sette A. (2006). Predicting population coverage of T-cell epitope-based diagnostics and vaccines. BMC Bioinform..

[18] Buchan D.W.A., Jones D.T. (2019). The PSIPRED Protein Analysis Workbench: 20 years on. Nucleic Acids Res..

[19] Ovchinnikov S., Park H., Kim D.E., DiMaio F., Baker D. (2018). Protein structure prediction using Rosetta in CASP12. Proteins.

[20] Gasteiger E., Hoogland C., Gattiker A., Wilkins M.R., Appel R.D., Bairoch A. (2005). Protein identification and analysis tools on the ExPASy server. The Proteomics Protocols Handbook.

[21] Magnan C.N., Randall A., Baldi P. (2009). SOLpro: Accurate sequence-based prediction of protein solubility. Bioinformatics.

[22] McGuffin L.J., Bryson K., Jones D.T. (2000). The PSIPRED protein structure prediction server. Bioinformatics.

[23] Wang S., Li W., Liu S., Xu J. (2016). RaptorX-Property: A web server for protein structure property prediction. Nucleic Acids Res..

[24] Heo L., Park H., Seok C. (2013). GalaxyRefine: Protein structure refinement driven by side-chain repacking. Nucleic Acids Res..

[25] Yan Y., Zhang D., Zhou P., Li B., Huang S.Y. (2017). HDOCK: A web server for protein-protein and protein-DNA/RNA docking based on a hybrid strategy. Nucleic Acids Res..

[26] López-Blanco J.R., Aliaga J.I., Quintana-Ortí E.S., Chacón P. (2014). iMODS: Internal coordinates normal mode analysis server. Nucleic Acids Res..

[27] Grote A., Hiller K., Scheer M., Münch R., Nörtemann B., Hempel D.C., Jahn D. (2005). JCat: A novel tool to adapt codon usage of a target gene to its potential expression host. Nucleic Acids Res..

[28] Rapin N., Lund O., Bernaschi M., Castiglione F. (2010). Computational immunology meets bioinformatics: The use of prediction tools for molecular binding in the simulation of the immune system. PLoS ONE.

[29] Craig D.B., Dombkowski A.A. (2013). Disulfide by Design 2.0: A web-based tool for disulfide engineering in proteins. BMC Bioinform..

[30] Yan Y., Tao H., He J., Huang S.Y. (2020). The HDOCK server for integrated protein-protein docking. Nat. Protoc..

[31] Seliger B., Ferrone S. (2020). HLA Class I Antigen Processing Machinery Defects in Cancer Cells-Frequency, Functional Significance, and Clinical Relevance with Special Emphasis on Their Role in T Cell-Based Immunotherapy of Malignant Disease. Methods Mol. Biol..

[32] Schein C.H., Ivanciuc O., Braun W. (2007). Bioinformatics approaches to classifying allergens and predicting cross-reactivity. Immunol. Allergy Clin. N. Am..

[33] Patil R., Das S., Stanley A., Yadav L., Sudhakar A., Varma A.K. (2010). Optimized hydrophobic interactions and hydrogen bonding at the target-ligand interface leads the pathways of drug-designing. PLoS ONE.

[34] Khade P.M., Scaramozzino D., Kumar A., Lacidogna G., Carpinteri A., Jernigan R.L. (2021). hdANM: A new comprehensive dynamics model for protein hinges. Biophys. J..

