# Supplementary material for: Immunoinformatics Strategy to Develop a Novel Universal Multiple Epitope-Based COVID-19 Vaccine
Source: Vaccines (Basel). 2023 Jun 12;11(6):1090. doi: 10.3390/vaccines11061090 (PMC10304668; doi:10.3390/vaccines11061090)
Supplement: Supplementary file 1 [file vaccines-11-01090-s001.zip › File S1.pdf]

Conservation of N-protein among various variants of SARS-Cov-2

Site 111-218

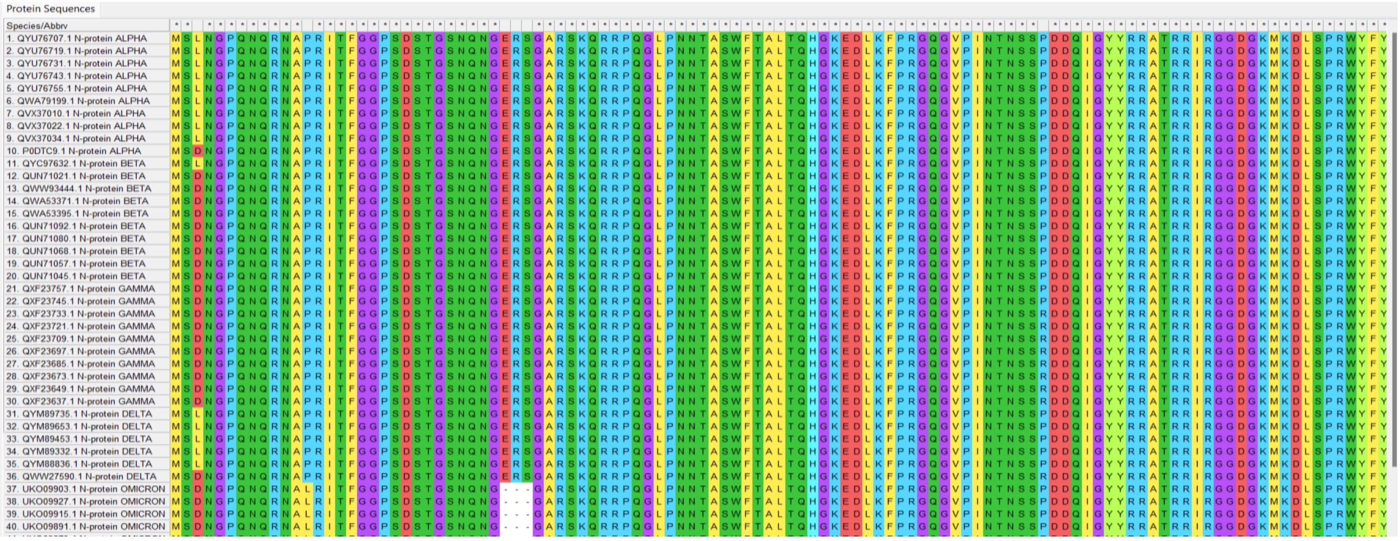

Site 111-219

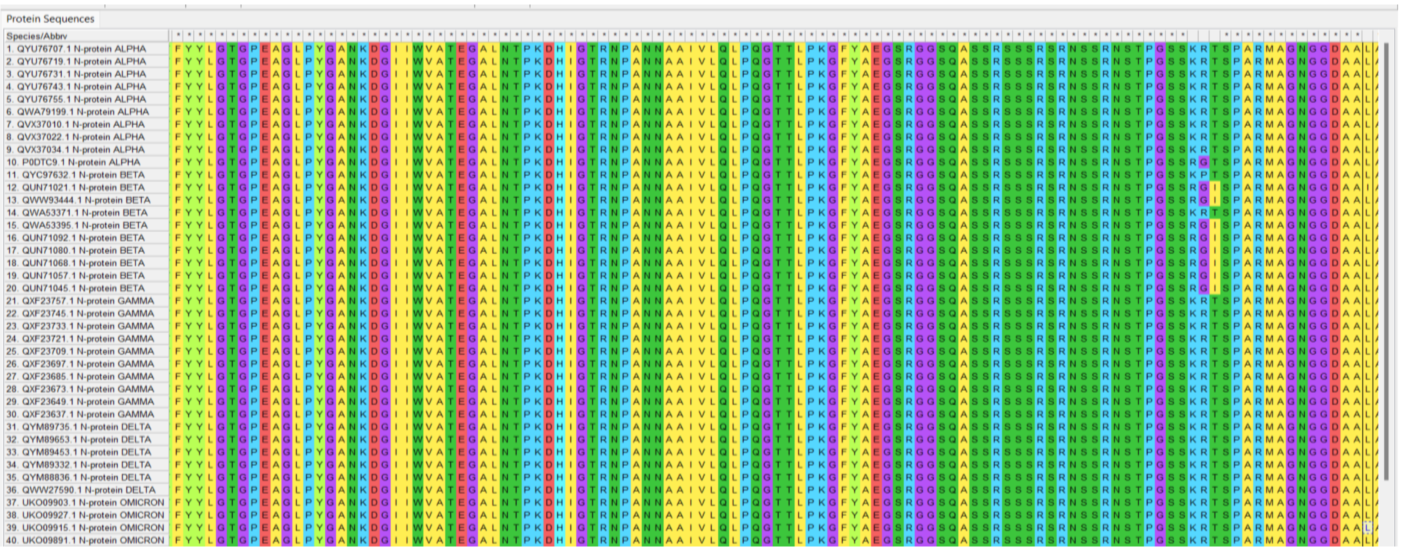

Site 111-219

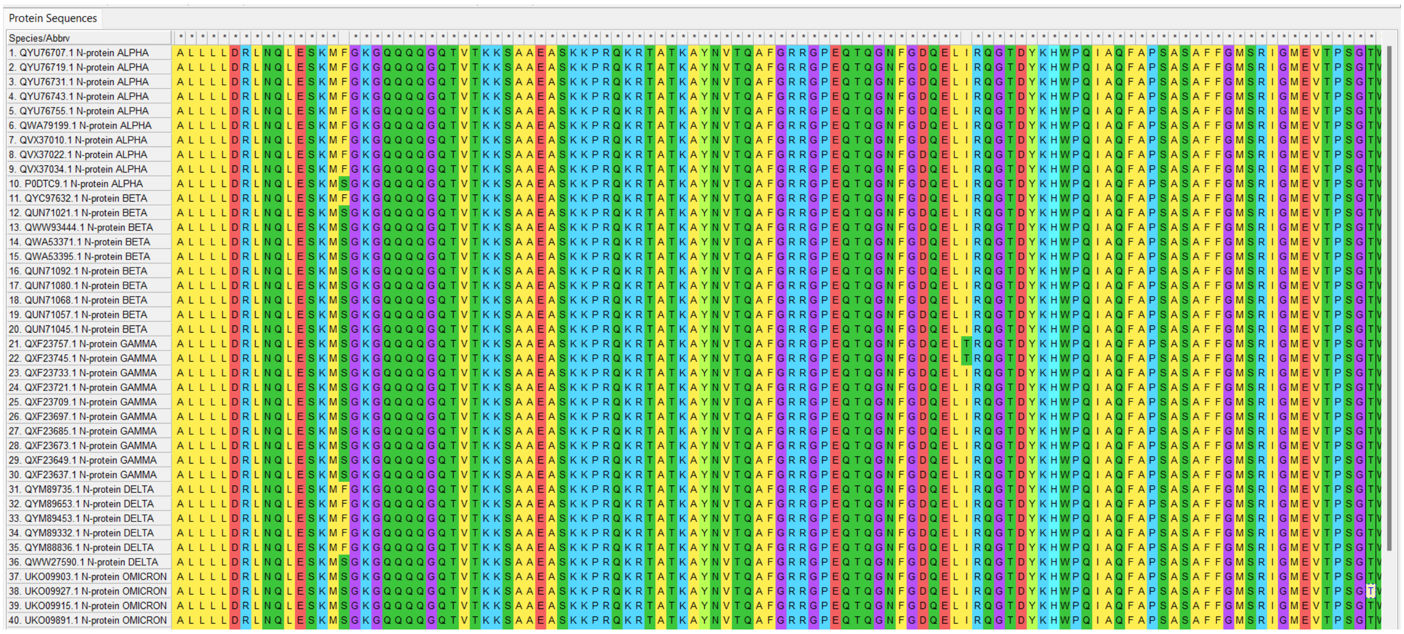

320 – 419

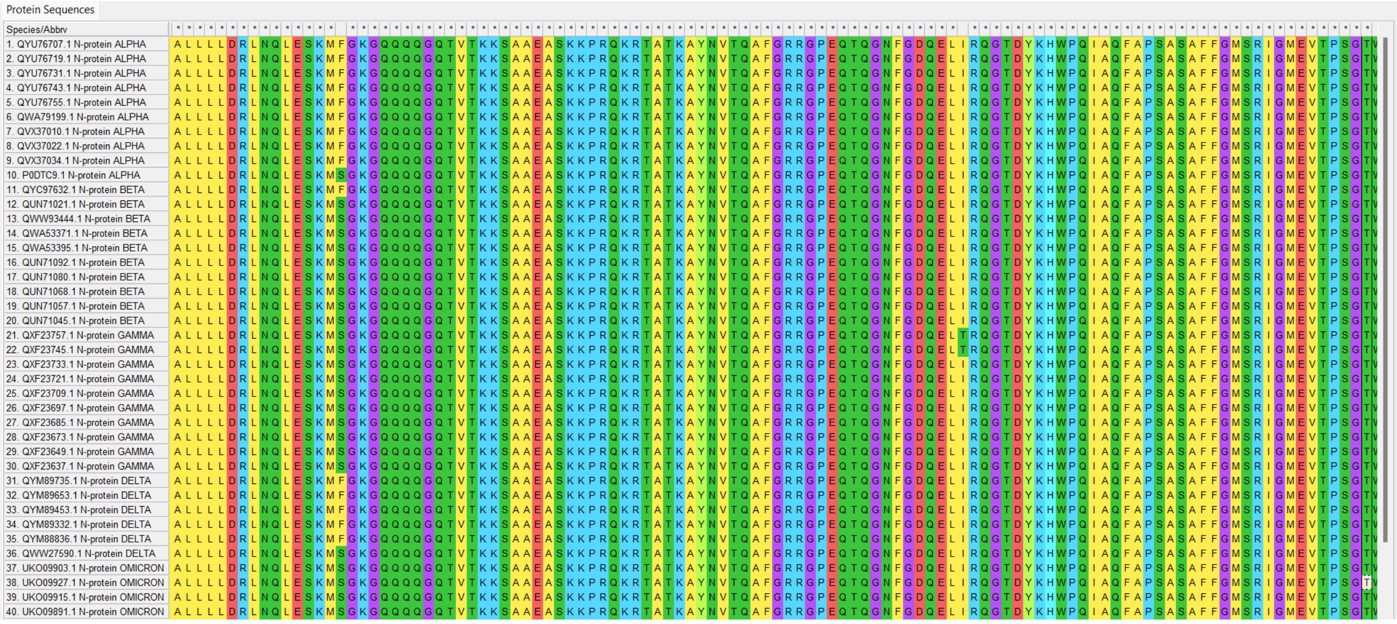
Protein Sequences
Species/Abbrv
1. QYU76707.1 N-protein ALPHA
2. QYU76719.1 N-protein ALPHA
3. QYU76731.1 N-protein ALPHA
4. QYU76743.1 N-protein ALPHA
5. QYU76755.1 N-protein ALPHA
6. QWA79199.1 N-protein ALPHA
7. QVX37010.1 N-protein ALPHA
8. QVX37022.1 N-protein ALPHA
9. QVX37034.1 N-protein ALPHA
10. P0DTC9.1 N-protein ALPHA
11. QYC97632.1 N-protein BETA
12. QUN71021.1 N-protein BETA
13. QWW93444.1 N-protein BETA
14. QWA53371.1 N-protein BETA
15. QWA53395.1 N-protein BETA
16. QUN71092.1 N-protein BETA
17. QUN71080.1 N-protein BETA
18. QUN71068.1 N-protein BETA
19. QUN71057.1 N-protein BETA
20. QUN71045.1 N-protein BETA
21. QXF23757.1 N-protein GAMMA
22. QXF23745.1 N-protein GAMMA
23. QXF23733.1 N-protein GAMMA
24. QXF23721.1 N-protein GAMMA
25. QXF23709.1 N-protein GAMMA
26. QXF23697.1 N-protein GAMMA
27. QXF23685.1 N-protein GAMMA
28. QXF23673.1 N-protein GAMMA
29. QXF23649.1 N-protein GAMMA
30. QXF23637.1 N-protein GAMMA
31. QYM89735.1 N-protein DELTA
32. QYM89653.1 N-protein DELTA
33. QYM89453.1 N-protein DELTA
34. QYM89332.1 N-protein DELTA
35. QYM88836.1 N-protein DELTA
36. QWW27590.1 N-protein DELTA
37. UKO09903.1 N-protein OMICRON
38. UKO09927.1 N-protein OMICRON
39. UKO09915.1 N-protein OMICRON
40. UKO09891.1 N-protein OMICRON
ALLLLDRLNQLESKMFGKGQQQQGQTVTKKSAAEASKKPRQKRTATKAYNVTQAFGRRGPEQTQGNFGDQELIRQGTDYKHWPQIAQFAPSASAFFGMSRIGMEVTPSGTW
