# Supplementary material for: Immunoinformatics Strategy to Develop a Novel Universal Multiple Epitope-Based COVID-19 Vaccine
Source: Vaccines (Basel). 2023 Jun 12;11(6):1090. doi: 10.3390/vaccines11061090 (PMC10304668; doi:10.3390/vaccines11061090)

1. Global Allelic coverage

IEDB Analysis Resource

Home | Help | Example | Reference | Download | Contact

Population Coverage Calculation Result

population/area	Class I		
	coverage[a]	average_hit[b]	pc90[c]
World	88.45%	1.43	0.87
**Average**	**88.45**	**1.43**	**0.87**
**Standard deviation**	**0.0**	**0.0**	**0.0**

[a] projected population coverage
[b] average number of epitope hits / HLA combinations recognized by the population
[c] minimum number of epitope hits / HLA combinations recognized by 90% of the population

Population: World

MHC class	Coverage	Average hit	PC90
I	88.45%	1.43	0.87

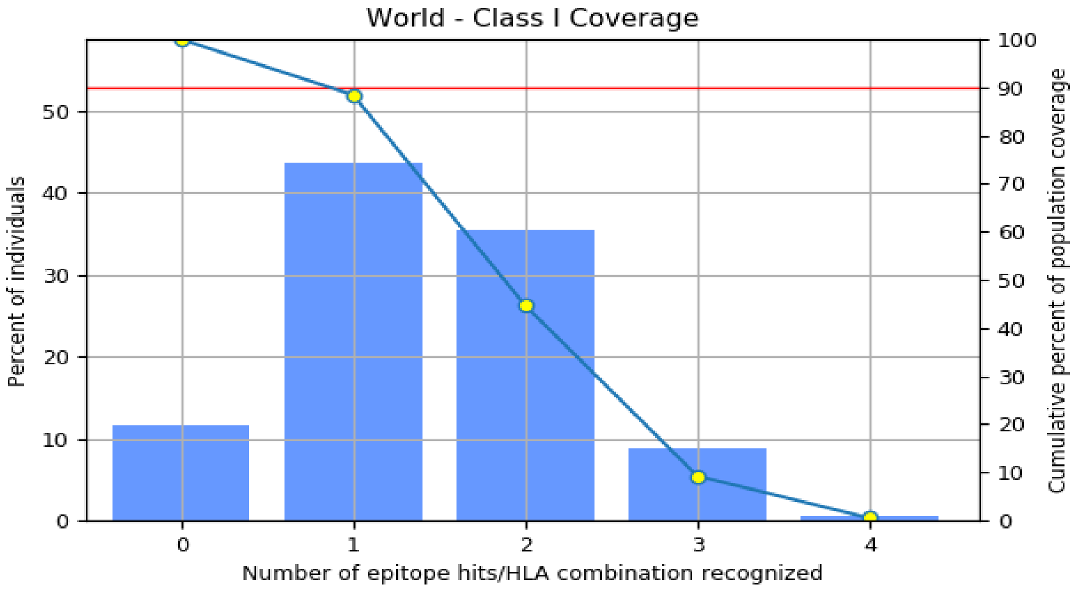

2. **Areawise Allelic coverage**

population/area	Class combined		
	coverage[a]	average_hit[b]	pc90[c]
Central Africa	69.7%	1.21	0.33
Central America	37.17%	0.51	0.16
East Africa	76.9%	1.42	0.43
East Asia	78.56%	1.85	0.47
Europe	97.9%	2.95	1.49
North Africa	87.36%	1.74	0.79
North America	94.7%	2.57	1.2
Northeast Asia	87.09%	1.86	0.77
Oceania	73.17%	1.17	0.37
South Africa	70.9%	1.01	0.34
South America	83.55%	1.74	0.61
South Asia	90.95%	2.22	1.03
Southeast Asia	79.12%	1.47	0.48
Southwest Asia	85.05%	1.59	0.67
West Africa	89.04%	2.16	0.91
West Indies	91.2%	2.2	1.04
**Average**	**80.77**	**1.73**	**0.69**
**Standard deviation**	**13.87**	**0.59**	**0.35**

[a] projected population coverage
[b] average number of epitope hits / HLA combinations recognized by the population
[c] minimum number of epitope hits / HLA combinations recognized by 90% of the population

3. Global Allelic coverage (Combined MHC clas-I and class-II)

population/area	Class combined		
	coverage[a]	average_hit[b]	pc90[c]
World	95.27%	2.62	1.25
**Average**	**95.27**	**2.62**	**1.25**
**Standard deviation**	**0.0**	**0.0**	**0.0**

[a] projected population coverage
[b] average number of epitope hits / HLA combinations recognized by the population
[c] minimum number of epitope hits / HLA combinations recognized by 90% of the population

Population: World

MHC class	Coverage	Average hit	PC90
combined	95.27%	2.62	1.25

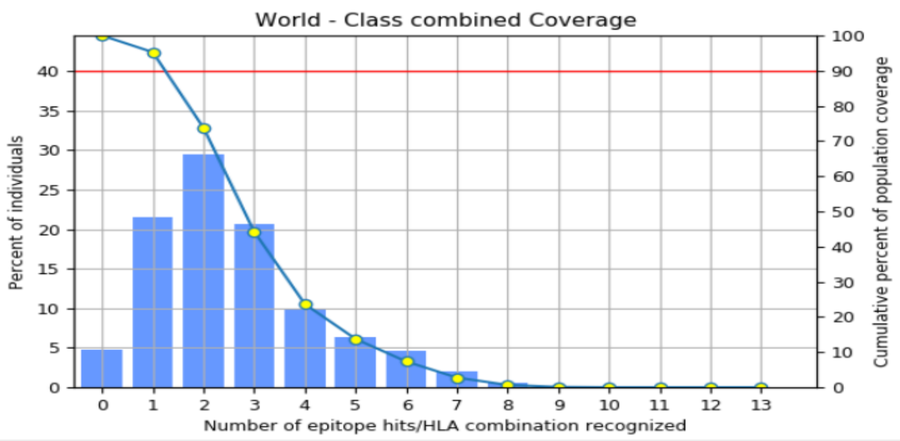

Supplement: Supplementary file 1 [file vaccines-11-01090-s001.zip › File S3.pdf]
