# Supplementary figures and images for: Immunoinformatics Strategy to Develop a Novel Universal Multiple Epitope-Based COVID-19 Vaccine
Source: Vaccines (Basel). 2023 Jun 12;11(6):1090. doi: 10.3390/vaccines11061090 (PMC10304668; doi:10.3390/vaccines11061090)

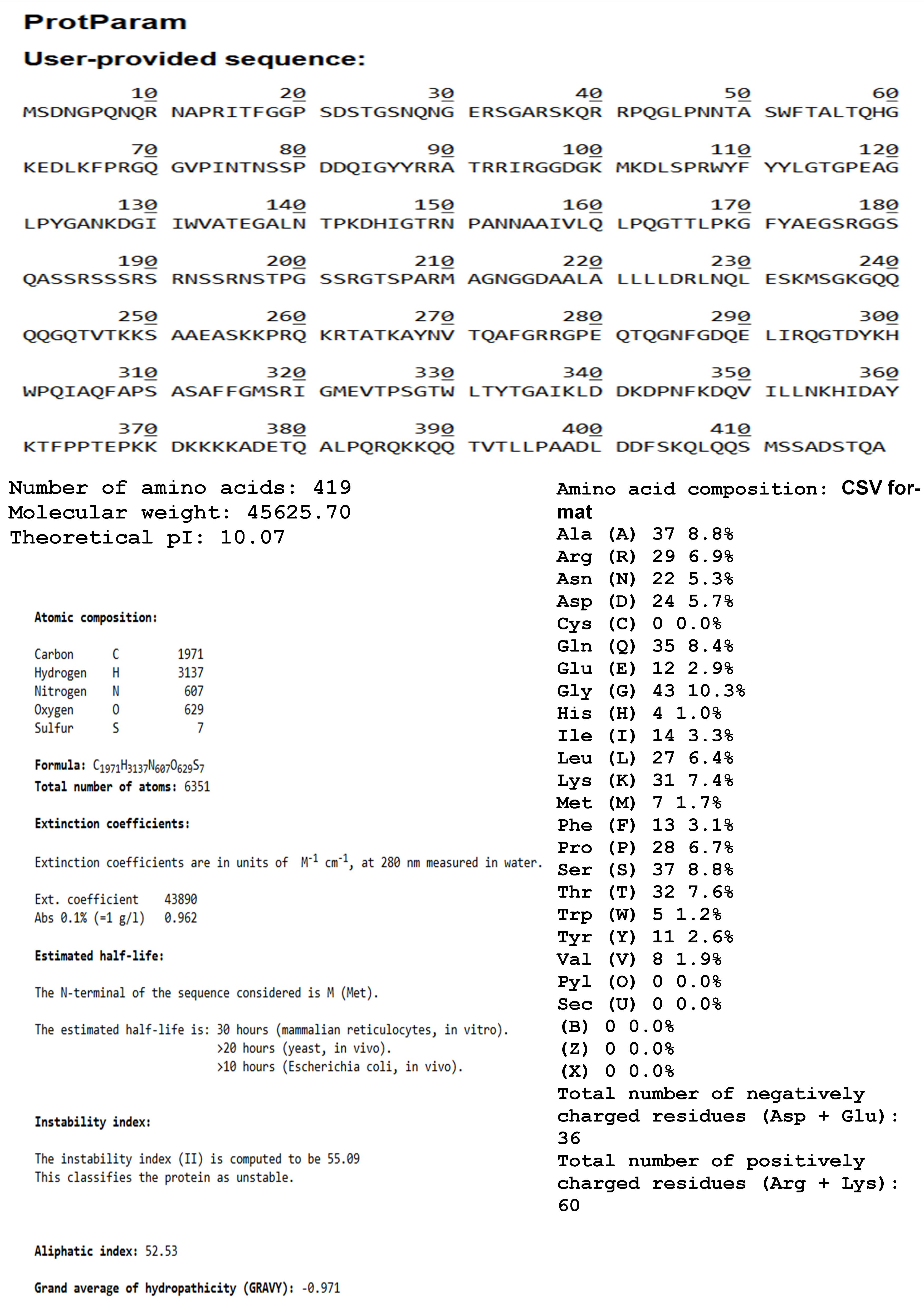

Supplement: Supplementary file 1 [file vaccines-11-01090-s001.zip › Figure S1.jpg]

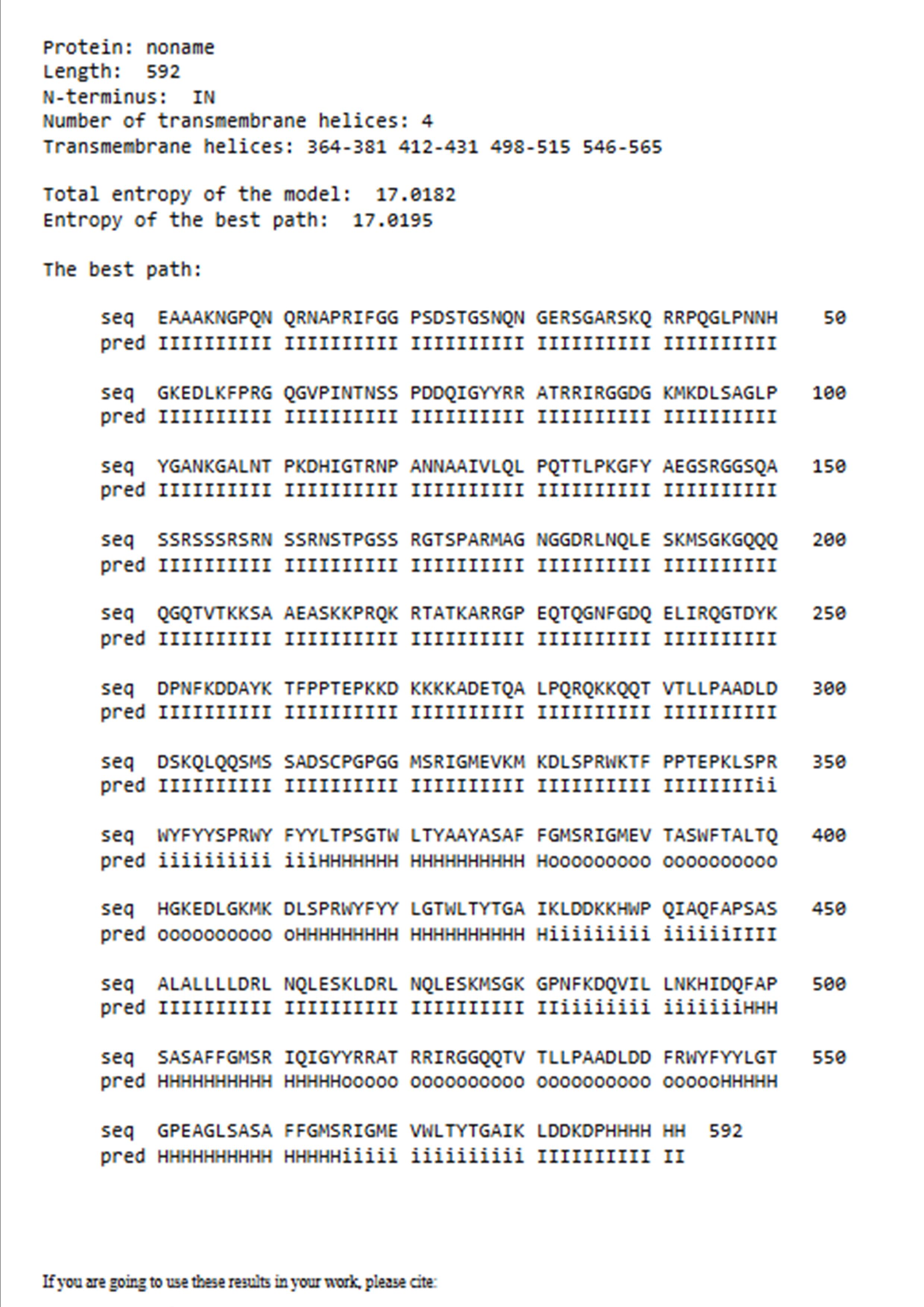

Supplement: Supplementary file 1 [file vaccines-11-01090-s001.zip › Figure S2.jpg]

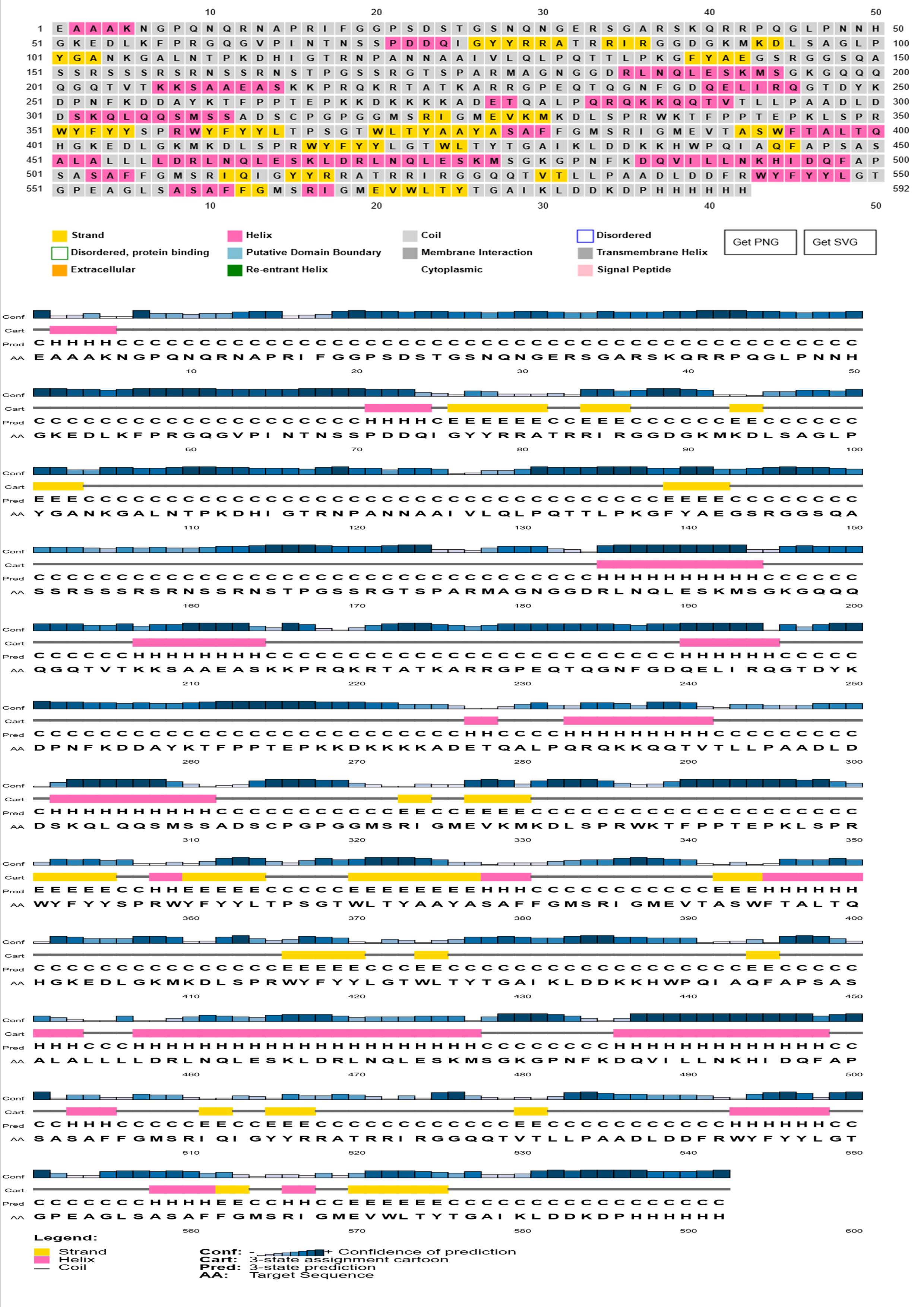

Supplement: Supplementary file 1 [file vaccines-11-01090-s001.zip › Figure S3.jpg]

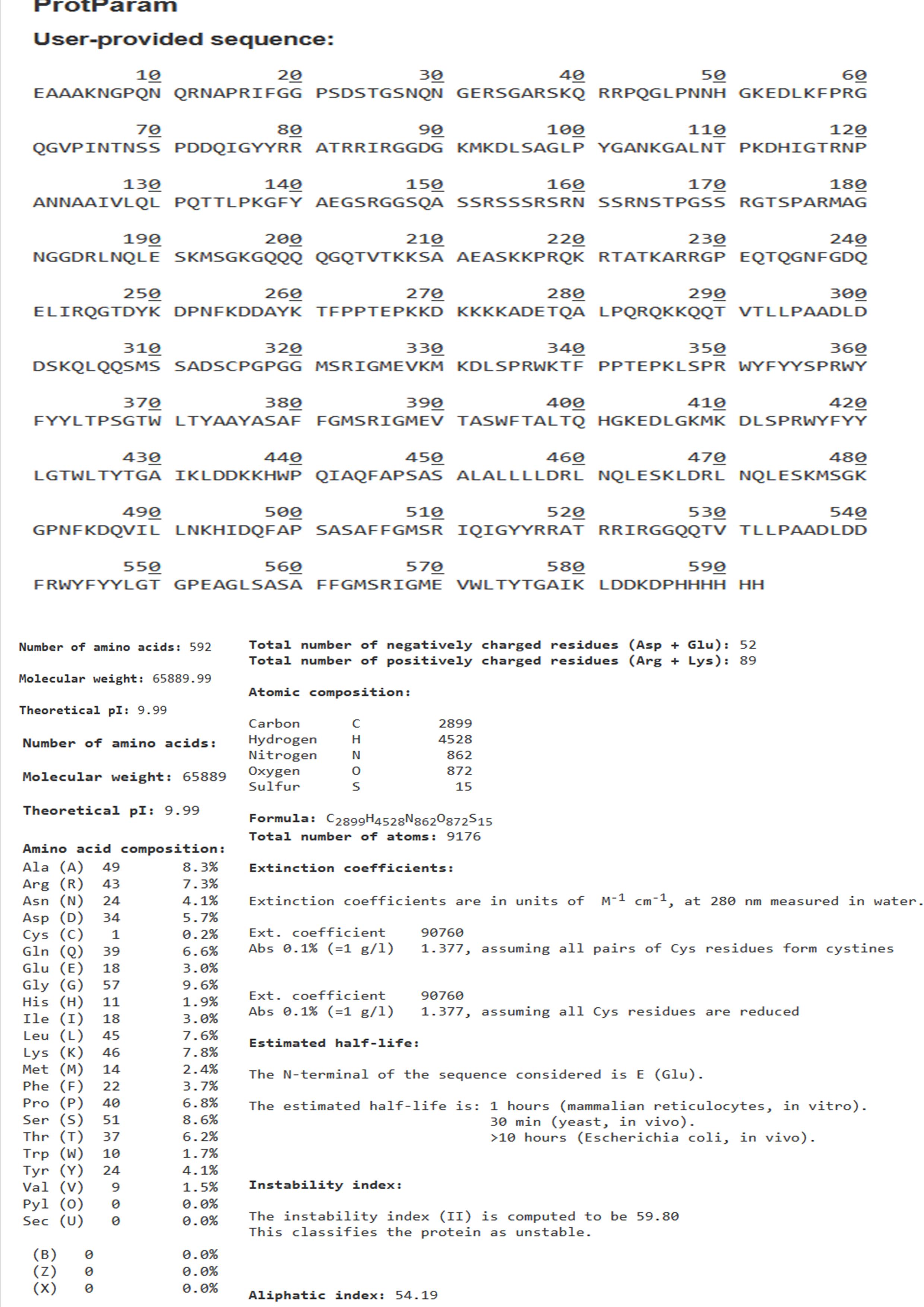

Supplement: Supplementary file 1 [file vaccines-11-01090-s001.zip › Figure S4.jpg]
